# DC Self-Field Critical Current in Superconductor/ Dirac-Cone Material/Superconductor Junctions

**DOI:** 10.3390/nano9111554

**Published:** 2019-11-01

**Authors:** Evgueni F. Talantsev

**Affiliations:** 1 M. N. Mikheev Institute of Metal Physics, Ural Branch, Russian Academy of Sciences, 18, S. Kovalevskoy St., Ekaterinburg 620108, Russia; evgeny.talantsev@imp.uran.ru or evgeny.talantsev@urfu.ru; Tel.: +7-912-676-0374; 2 NANOTECH Centre, Ural Federal University, 19 Mira St., Ekaterinburg 620002, Russia

**Keywords:** the self-field critical current, induced superconductivity in Dirac-cone materials, single layer graphene, multiple-band superconductivity

## Abstract

Recently, several research groups have reported on anomalous enhancement of the self-field critical currents, *I_c_*(sf,*T*), at low temperatures in superconductor/Dirac-cone material/superconductor (S/DCM/S) junctions. Some papers attributed the enhancement to the low-energy Andreev bound states arising from winding of the electronic wave function around DCM. In this paper, *I_c_*(sf,*T*) in S/DCM/S junctions have been analyzed by two approaches: modified Ambegaokar-Baratoff and ballistic Titov-Beenakker models. It is shown that the ballistic model, which is traditionally considered to be a basic model to describe *I_c_*(sf,*T*) in S/DCM/S junctions, is an inadequate tool to analyze experimental data from these type of junctions, while Ambegaokar-Baratoff model, which is generally considered to be a model for *I_c_*(sf,*T*) in superconductor/insulator/superconductor junctions, provides good experimental data description. Thus, there is a need to develop a new model for self-field critical currents in S/DCM/S systems.

## 1. Introduction

Intrinsic superconductors [1] of rectangular cross-section (with width 2*a* and thickness 2*b*) exhibit non-dissipative temperature dependent transport self-field critical current, *I_c_*(sf,*T*) (i.e., when no external magnetic field applies), which is given by the following universal equation [2,3,4]:

(1) $$I_{c}\left(\mathrm{sf},T\right)=\frac{\phi_{0}}{\pi \cdot \mu_{0}}\cdot \left[\frac{ln\left(1+\sqrt{2}\cdot \kappa_{c}\left(T\right)\right)}{\lambda_{ab}^{3}\left(T\right)}\cdot \left(\frac{\lambda_{c}\left(T\right)}{b}\cdot \tanh\left(\frac{b}{\lambda_{c}\left(T\right)}\right)\right)+\;\frac{ln\left(1+\sqrt{2}\cdot \gamma \left(T\right)\cdot \kappa_{c}\left(T\right)\right)}{\sqrt{\gamma \left(T\right)}\cdot \lambda_{ab}^{3}\left(T\right)}\left(\frac{\lambda_{ab}\left(T\right)}{a}\tanh\left(\frac{a}{\lambda_{ab}\left(T\right)}\right)\right)\right]\cdot \left(a\cdot b\right),$$

where *T* is sample temperature, $\phi_{0}=2.067\times 10^{-15}\;Wb$ is the magnetic flux quantum, $\mu_{0}=4\cdot \pi \times 10^{-7}\;H/m$ is the magnetic permeability of free space, $\lambda_{ab}\left(T\right)$ and $\lambda_{c}\left(T\right)$ are the in-plane and out-of-plane London penetration depths respectively, $\kappa_{c}\left(T\right)=\lambda_{ab}\left(T\right)/\xi_{ab}\left(T\right)$, $\xi_{ab}\left(T\right)$ is the in-plane coherence length, and $\gamma \left(T\right)=\lambda_{c}\left(T\right)/\lambda_{ab}\left(T\right)$ is the electron mass anisotropy. It has been shown in previous research that Equation (1) quantitatively and accurately describes *I_c_*(sf,*T*) in more than 100 superconductors, ranging from elemental Zn with *T_c_* = 0.65 K to highly-compressed H_3_S with $T_{c}≳200\;K$ [2,3,4], and samples dimensions from several Å to about 1 mm [5].

All intrinsic superconductors [1] can induce a superconducting state in non-superconducting materials by the Holm-Meissner effect [6]. However, a universal equation for non-dissipative self-field critical transport current, *I_c_*(sf,*T*), in superconductor/non-superconductor/superconductor junctions is still unknown. Ambegaokar and Baratoff (AB) [7,8] were the first who proposed an equation for *I_c_*(sf,*T*) in superconductor/insulator/superconductor (S/I/S) systems [9]. Later, Kulik and Omel’yanchuk (KO) [10,11,12] proposed two models for different types of superconductor/normal conductor/superconductor junctions (which are known as KO-1 [10] and KO-2 [11]).

In general, superconductor/normal metal/superconductor (S/N/S) junctions are classified by the comparison of the device length (*L*) to two characteristic length scales of the junction, which are the mean free path of the charge carriers, *l*_e_, and the superconducting correlation length, ξ*_s_*. These length scales classify whether the junction is in short (*L* ≪ ξ*_s_*) or long (i.e., *L* ≫ ξ*_s_*) regime and ballistic (*L* ≪ *l*_e_) or diffusive (*L* ≫ *l_e_*) limit, respectively.

For about one decade, the KO-1 model was considered to be the primary model to describe *I_c_*(sf,*T*) in superconductor/graphene/superconductor (S/G/S) junctions (a detailed review of different models for *I_c_*(sf,*T*) in S/G/S junctions was given by Lee and Lee [13]). However, recent technological progress in fabricating high-quality S/G/S junctions demonstrates a large difference between the KO-1 model and experimental *I_c_*(sf,*T*) data [14]. A detailed discussion of all models, including a model by Takane and Imura [15], which was proposed to describe *I_c_*(sf,*T*) in superconductor/Dirac-cone material/superconductor (S/DCM/S) junctions, is given by Lee and Lee [13].

It should be noted that a universal quantitatively accurate equation for critical currents at the applied magnetic field, *B*, is unknown to date for intrinsic superconductors [16,17,18,19,20] and for Josephson junctions [13,21,22]. However, the discussion of these important problems, as well as the discussion of interface superconductivity [23,24,25] and generic case of two-dimensional (2D) superconductivity [26,27,28,29,30,31,32,33,34,35,36,37,38,39,40,41,42,43,44,45,46,47,48,49,50], is beyond the scope of this paper.

The primary task for this work is to show that *I_c_*(sf,*T*), in a variety of S/DCM/S junctions in the ballistic regime, cannot be described by the KO-based model. To prove this, experimental *I_c_*(sf,*T*) datasets in S/DCM/S junctions were analyzed by two models: the modified Ambegaokar-Baratoff model [51,52] and ballistic Titov-Beenakker model [53].

It needs to be noted that some S/DCM/S junctions show the *I_c_*(sf,*T*) enhancement at a reduced temperature of *T* ≤ 0.25·*T_c_*. For instance, the enhancement in atomically-thin MoRe/single layer graphene (SLG)/MoRe junction was first reported by Calado et al. [54]. Raw experimental *I_c_*(sf,*T*) data reported by Borzenets et al. [55] in nominally the same MoRe/SLG/MoRe junctions also shows the enhancement at *T* ≤ 0.25·*T_c_*. Based on this, the *I_c_*(sf,*T*) enhancement at low reduced temperatures in Nb/BiSbTeSe_2_-nanoribon/Nb reported by Kayyalha et al. [56] cannot be considered as a unique property of superconductor/topological insulator/superconductor (S/TI/S) junctions, but is rather the demonstration of a general feature of S/DCM/S devices and atomically thin superconducting systems. Additionally, it is important to mention that Kurter et al. [57] were the first who reported *I_c_*(sf,*T*) enhancement in S/TI-nanoribbon/S junction at reduced temperature of *T* ≤ 0.25·*T*_c_.

As a result of the performed *I_c_*(sf,*T*) analysis in this paper, it is shown that a new model is needed to describe dissipation-free transport currents in S/DCM/S junctions.

## 2. Models Description

The amplitude of dissipation-free transport current, *I_c_*(sf,*T*), in S/I/S junction was first given by Ambegaokar and Baratoff (AB) [7,8]:

(2) $$I_{c}\left(\mathrm{sf},T\right)=\frac{\pi \cdot \Delta \left(T\right)}{2\cdot e\cdot R_{n}}\cdot \tanh\left(\frac{\Delta \left(T\right)}{2\cdot k_{B}\cdot T}\right),$$

where ∆(*T*) is the temperature-dependent superconducting gap, *e* is the electron charge, *R_n_* is the normal-state tunneling resistance in the junction, and *k_B_* is the Boltzmann constant. In one research [51], it was proposed to substitute ∆(*T*) in Equation (2) by the analytical expression given by Gross et al. [58]:

(3) $$\Delta \left(T\right)=\Delta \left(0\right)\cdot \tanh\left(\frac{\pi \cdot k_{B}\cdot T_{c}}{\Delta \left(0\right)}\cdot \sqrt{\eta \cdot \left(\frac{\Delta C}{C}\right)\cdot \left(\frac{T_{c}}{T}-1\right)}\right),$$

where Δ(0) is the ground-state amplitude of the superconducting band, Δ*C*/*C* is the relative jump in electronic specific heat at the transition temperature, *T_c_*, and *η* = 2/3 for *s*-wave superconductors [56]. In the result, *T_c_*, Δ*C*/*C*, Δ(0), and normal-state tunneling resistance, *R*_n_, of the S/I/S junction, or in the more general case of S/N/S junction, can be deduced by fitting experimental *I_c_*(sf,*T*) datasets to Equation (2), for which the full expression is [51]:

(4) $$I_{c}\left(\mathrm{sf},T\right)=\frac{\pi \cdot \Delta \left(0\right)\cdot \tanh\left(\frac{\pi \cdot k_{B}\cdot T_{c}}{\Delta \left(0\right)}\cdot \sqrt{\eta \cdot \left(\frac{\Delta C}{C}\right)\cdot \left(\frac{T_{c}}{T}-1\right)}\right)}{2\cdot e\cdot R_{n}}\cdot \;\tanh\left(\frac{\Delta \left(0\right)\cdot \tanh\left(\frac{\pi \cdot k_{B}\cdot T_{c}}{\Delta \left(0\right)}\cdot \sqrt{\eta \cdot \left(\frac{\Delta C}{C}\right)\cdot \left(\frac{T_{c}}{T}-1\right)}\right)}{2\cdot k_{B}\cdot T}\right),$$

It should be noted that direct experiments performed by Natterer et al. [59] showed that the superconducting gap does exist in graphene, which is in proximity contact with superconducting electrodes. The gap amplitude, Δ(*T*), has a characteristic decaying length [59], which is the expected behavior from primary idea of the proximity effect [6]. As a direct consequence, clear physical meaning remains for the relative jump in electronic specific heat at the transition temperature, Δ*C*/*C*, due to this parameter is an essential thermodynamic consequence for the appearance of the superconducting energy gap, Δ(*T*). As was shown in another study [51], Δ*C*/*C* is the fastest decaying parameter of the superconducting state in S/N/S junctions, over the junction length, *L*, while *T*_c_ is the most robust one.

In References [51,52], it was shown that S/SLG/S and S/Bi_2_Se_3_/S junctions exhibit two-decoupled band superconducting state. Thus, for the general case of *N*-decoupled bands, the temperature-dependent self-field critical current, *I_c_*(sf,*T*), can be described by the following equation:

(5) $$I_{c}\left(\mathrm{sf},T\right)=\sum_{i=1}^{N}\frac{\pi \cdot \Delta_{i}\left(T\right)}{2\cdot e\cdot R_{n,i}}\cdot \theta \left(T_{c,i}-T\right)\cdot \tanh\left(\frac{\Delta_{i}\left(T\right)}{2\cdot k_{B}\cdot T}\right),$$

where the subscript *i* refers to the *i*-band, *θ*(*x*) is the Heaviside step function, and each band has its own independent parameters of *T_c_*_,*i*_, Δ*C_i_*/*C_i_*, Δ*_i_*(0), and *R_n_*_,*i*_. Equation (5) was also used to analyze experimental *I_c_*(sf,*T*) data for several S/DCM/S junctions [60]. 

Titov and Beenakker [53] proposed that *I_c_*(sf,*T*) in S/DCM/S junction at the conditions near the Dirac point can be described by the equation:

(6) $$I_{c}\left(\mathrm{sf},T\right)=1.33\cdot \frac{e\cdot \Delta \left(T\right)}{\hbar }\cdot \frac{W}{\pi \cdot L},$$

where *W* is the junction width. In this paper, analytical equation for the gap (Equation (3) [57]) is substituted in Equation (6):

(7) $$I_{c}\left(\mathrm{sf},T\right)=1.33\cdot \frac{e\cdot \Delta \left(0\right)\cdot \tanh\left(\frac{\pi \cdot k_{B}\cdot T_{c}}{\Delta \left(0\right)}\cdot \sqrt{\eta \cdot \left(\frac{\Delta C}{C}\right)\cdot \left(\frac{T_{c}}{T}-1\right)}\right)}{\hbar }\cdot \frac{W}{\pi \cdot L},$$

with the purpose to deduce *T*_c_, Δ*C*/*C*, and Δ(0) values in the S/DCM/S junctions from the fit of experimental *I_c_*(sf,*T*) datasets to Equation (7). For a general case of *N*-decoupled bands, temperature-dependent self-field critical current *I_c_*(sf,*T*) in S/DCM/S junctions can be described by the following equation:

(8) $$I_{c}\left(\mathrm{sf},T\right)=1.33\cdot \frac{e}{\pi \cdot \hbar }\cdot \frac{W}{L}\cdot \sum_{i=1}^{N}\Delta_{i}\left(T\right)\cdot \theta \left(T_{c,i}-T\right),$$

Based on a fact that *W* and *L* can be measured with very high accuracies, Equation (7) has the minimal ever proposed number of free-fitting parameters (which are *T_c_*, Δ*C*/*C*, Δ(0)) to fit to the experimental *I_c_*(sf,*T*) dataset. However, as we demonstrate below, the ballistic model (Equation (6) [53]) is not the most correct model to describe *I_c_*(sf,*T*) in S/DCM/S junctions. It should be noted that Equation (4) utilizes the same minimal set of parameters within the Bardeen-Cooper-Schrieffer (BCS) theory [60], i.e., *T_c_*, Δ*C*/*C*, Δ(0), to describe superconducting state in S/N/S junction and *R_n_* as a free-fitting parameter to describe the junction.

It should be stressed that a good reason must be presented for requiring a more complex model than is needed to adequately explain the experimental data [61,62].

In the next section, Equations (4), (5), (7), and (8) will be applied to fit experimental *I_c_*(sf,*T*) datasets for a variety of S/DCM/S junctions with the purpose to reveal the primary superconducting parameters of these systems and by comparison deduced parameters with weak-coupling s-wave BCS limits we show that the modified Ambegaokar and Baratoff model (Equations (4) and (5)) [51,52] describes the superconducting state in S/DCM/S junctions with higher accuracy.

## 3. Results

### 3.1. Micrometer-Long Tantalum/Graphene/Tantalum (Ta/G/Ta) Junction

**Figure 1:**
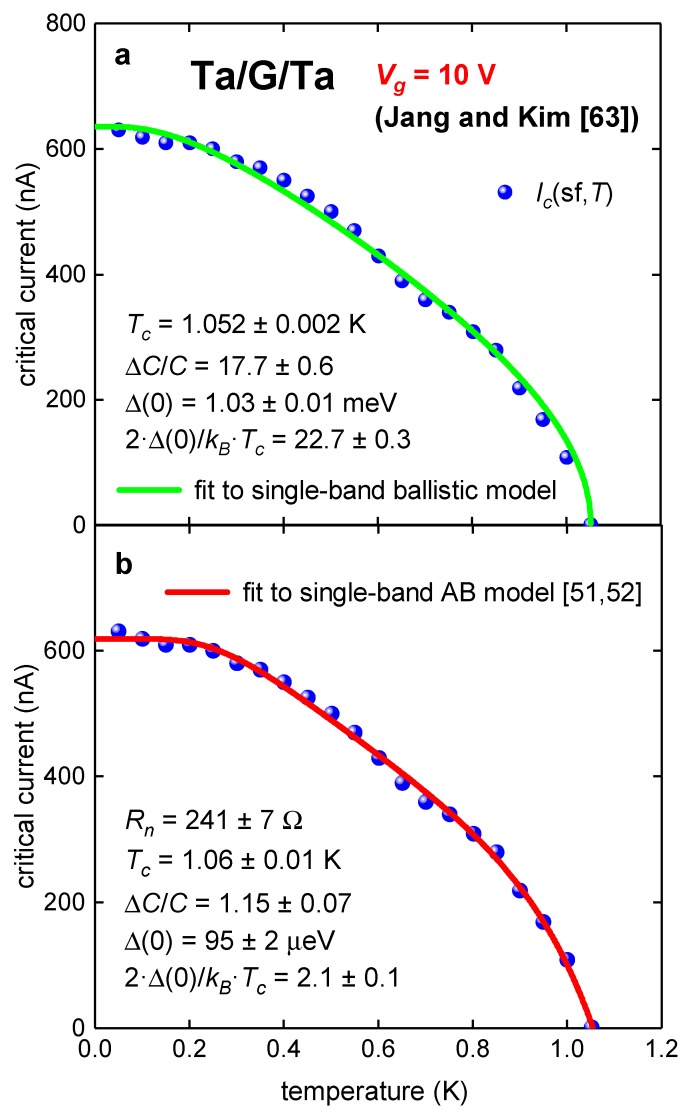
Experimental *I_c_(sf*,*T*) for tantalum/graphene/tantalum (Ta/G/Ta) junction (Device 1) at gate voltage of *V_g_* = 10 V [63] and data fits to single-band ballistic model (Equation (7), Panel a) and single-band modified AB model (Equation (4), Panel b) (**a**) Ballistic model. fit quality is *R* = 0.9948; (**b**) modified AB model [51,52] fit quality is *R* = 0.9980.

**Figure 2:**
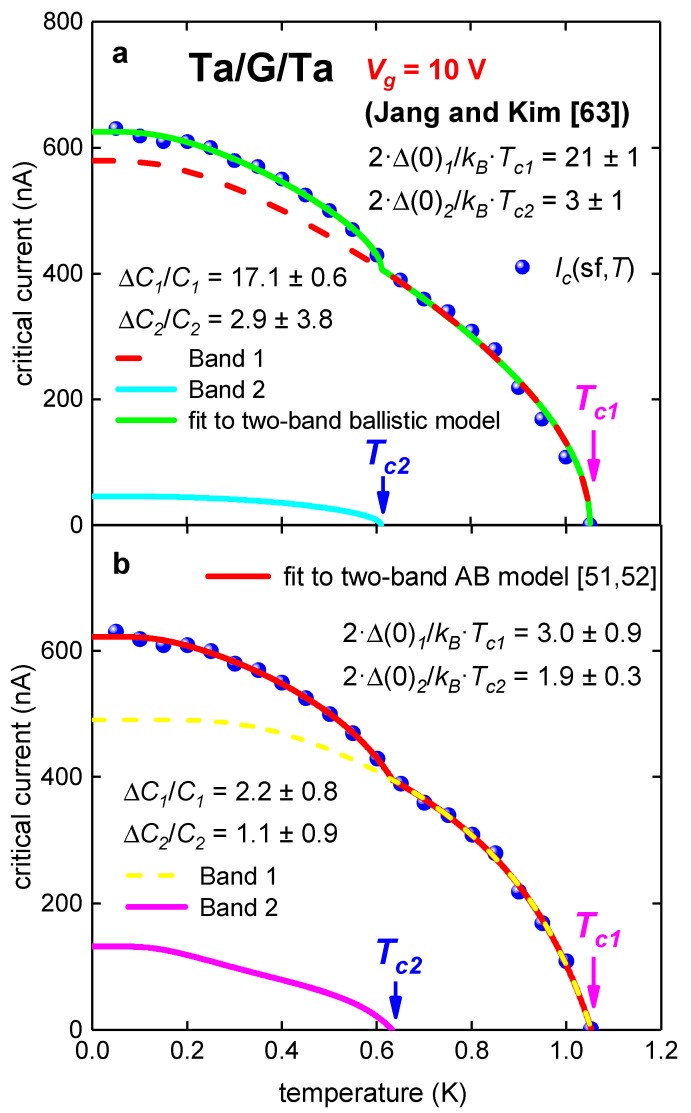
Experimental *I_c_*(sf,*T*) for Ta/G/Ta junction (Device 1) at gate voltage of *V_g_* = 10 V [63] and data fits to two-band ballistic model (Equation (8), Panel a) and two-band modified AB model (Equation (5), Panel b). (**a**) Ballistic model, fit quality is *R* = 0.9978; (**b**) modified AB model [51,52]. Derived parameters: *R_n1_* = 429 ± 184 Ω, *R_n2_* = 603 ± 209 Ω, fit quality is *R* = 0.9994.

Jang and Kim [63] reported experimental *I_c_*(sf,*T*) datasets and fit to KO-1 model (in their Figure 2d [63]) for micrometer long ballistic Ta/G/Ta junctions. The *I_c_*(sf,*T*) fit to KO-1 model (Figure 2d [63]) and deduced parameters are in disagreement with experimental values based on *I_c_R_n_* product. In Figure 1, we show *I_c_*(sf,*T*) datasets for Device 1 [63] (recorded at gate voltage *V_g_* = 10 V) and fits to single-band ballistic model, Equation (7) (in Figure 1a) and single-band modified AB model Equation (4) (Figure 1b). Device 1 has *W* = 6 µm, *L* = 1 µm, and ξ*_s_* = 16 µm [63]. This means that the ballistic limit of *L* << ξ*_s_* is satisfied for these junctions.

Results of fits to both models are presented in Table 1.

Deduced parameters from the fit to ballistic model (Equation (7)) in Figure 1a are in remarkable disagreement with any physical-backgrounded expectations, i.e., the ratio of $\frac{2\cdot \Delta \left(0\right)}{k_{B}\cdot T_{c}}=22.7$ (which should be comparable with *s*-wave BCS weak coupling limit of $\frac{2\cdot \Delta \left(0\right)}{k_{B}\cdot T_{c}}=3.53$) and $\frac{\Delta C}{C}=17.7$ (which should be comparable with *s*-wave BCS weak coupling limit of $\frac{\Delta C}{C}=1.43$).

It needs to be noted that the highest experimental value for phonon-mediated superconductors of $\frac{2\cdot \Delta \left(0\right)}{k_{B}\cdot T_{c}}\approx 5$ was measured for lead- and bismuth-based alloys [64,65], and the deduced value by the ballistic model $\frac{2\cdot \Delta \left(0\right)}{k_{B}\cdot T_{c}}\approx 23$ does not have a physical interpretation.

In contract, the fit to Equation (4) reveals superconducting parameters in expected ranges of $\frac{2\cdot \Delta \left(0\right)}{k_{B}\cdot T_{c}}=2.1\pm 0.1$ and $\frac{\Delta C}{C}=1.15\pm 0.07$, i.e., these parameters are slightly suppressed from *s*-wave BCS weak-coupling limits as expected [52,60]. It should also be noted that free-fitting parameter *R_n_* = 241 ± 7 Ω is in a good agreement with experimental measured value for this junction [63].

It can be seen (Figure 1), that there is an upturn in experimental *I_c_*(sf,*T*) at *T* ~ 0.65 K, which is a manifestation of the second superconducting band opening in this atomically thin S/N/S junction [51,52]. Thus, the experimental *I_c_*(sf,*T*) dataset was fitted to two-band models (Equations (8) and (5)). Results of these fits are shown in Figure 2 and deduced parameters are in Table 2.

The fit reveals a large disagreement of parameters deduced by ballistic model with expected values within frames for BCS theory. In contrast with this, deduced parameters by modified AB model [51,52] are within weak-coupling limits of BCS. As shown in Reference [51], raw experimental *I_c_*(sf,*T*) datasets should be reasonably dense to deduce parameters by AB model with small uncertainties.

### 3.2. Planar Nb/BiSbTeSe_2_-Nanoribbon/Nb Junctions

**Figure 3:**
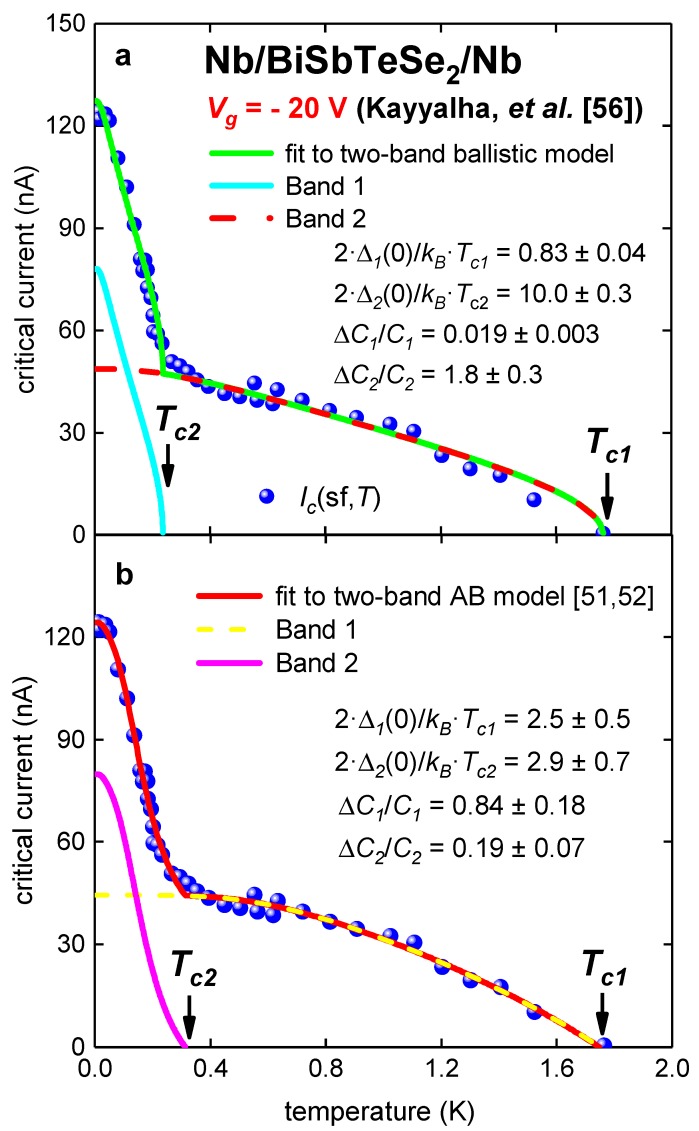
Experimental *I_c_*(sf,*T*) for Nb/BiSbTeSe_2_-nanoribbon/Nb junction (Sample 1 [56]) at gate voltage *V_g_* = −20 V. (**a**) Ballistic model, fit quality is *R* = 0.990; (**b**) modified AB model [51,52]. Derived parameters: *R_n1_* = 6.7 ± 1.6 kΩ, *R_n2_* = 0.75 ± 0.18 kΩ, fit quality is *R* = 0.9953.

Kayyalha et al. [56] reported *I_c_*(sf,*T*) for five Nb/BiSbTeSe_2_-nanopribbon/Nb junctions at different gate voltage, *V_g_*. In this paper *I_c_*(sf,*T*) datasets for Sample 1 at *V_g_* = −20 V, 0 V and 45 V [56] were analyzed by two-band models (Equations (5) and (8)), because it was already shown in Reference [60] that these junctions exhibit two-band superconducting state. In Figure 3 experimental *I_c_*(sf,*T*) dataset [56] and fits are shown. For this junction, *L* = 40 nm [56] and ξ*_s_* = 640 nm [56]; thus, the ballistic regime, *L* << ξ*_s_*, is well satisfied.

Despite the fact that fits to both models have a similar quality, deduced parameters of the superconducting state (Table 3), i.e., Δ*C_i_*/*C_i_*, Δ*_i_*(0), and $\frac{2\cdot \Delta_{i}\left(0\right)}{k_{B}\cdot T_{c,i}}$, for the case of the ballistic models (Figure 3a), similar to the case of Ta/G/Ta junction (Figure 1 and Figure 2), are remarkably different from values expected from BCS theory. Additionally, there are two orders of magnitude difference between deduced Δ*C_i_*/*C_i_* for two bands for the same sample, and one order of magnitude for $\frac{2\cdot \Delta_{i}\left(0\right)}{k_{B}\cdot T_{c,i}}$, which is unavoidable evidence that the ballistic model needs to be reexamined. In contrast with this, the fit to the modified AB model [51] (Figure 3b) reveals deduced parameters, including *R_ni_* values, in the expected ranges. It should be noted that full analysis (within the modified AB model [52]) of *I_c_*(sf,*T*) datasets in junctions reported by Kayyalha et al. [56] can be found elsewhere [60].

**Figure 4:**
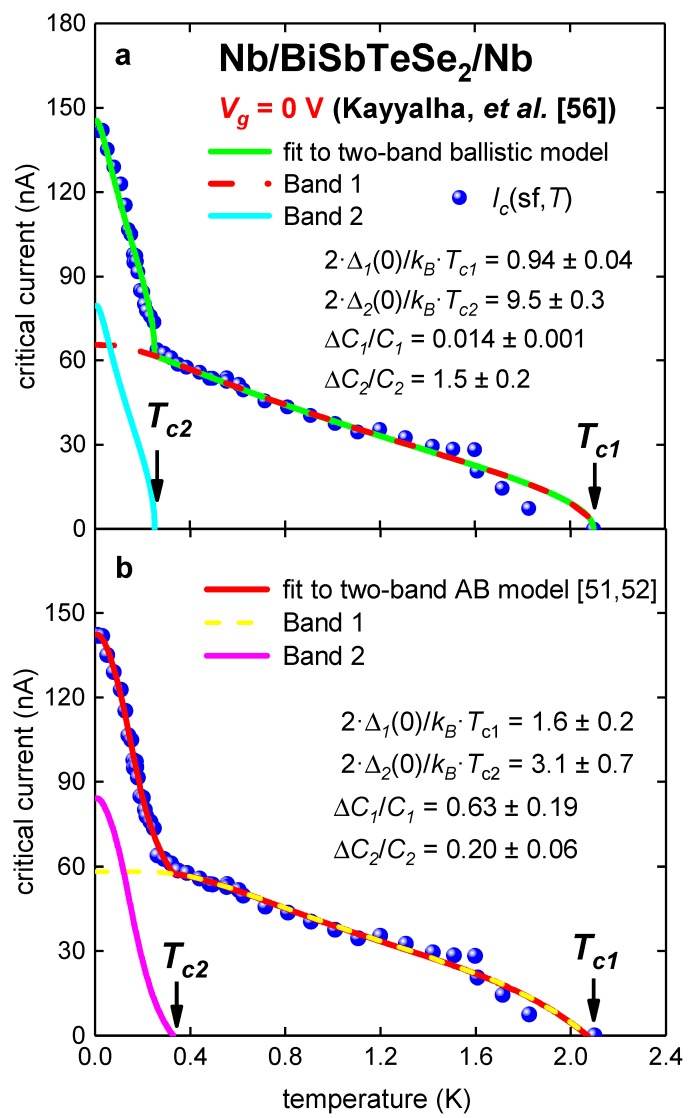
Experimental *I_c_*(sf,*T*) for Nb/BiSbTeSe_2_-nanoribbon/Nb junction (Sample 1 [56]) at gate voltage *V_g_* = 0 V. (**a**) Ballistic model, fit quality is *R* = 0.992; (**b**) modified AB model [51,52]. Derived parameters: *R_n1_* = 3.9 ± 0.4 kΩ, *R_n2_* = 0.81 ± 0.15 kΩ, fit quality is *R* = 0.9965.

In Figure 4, experimental *I_c_*(sf,*T*) dataset [56] and fits to two models for Sample 1 at gate voltage *V_g_* = 0 V also demonstrate that the ballistic model is an inadequate tool to analyze experimental data in S/DCM/S junctions (deduced parameters are given in Table 4).

**Figure 5:**
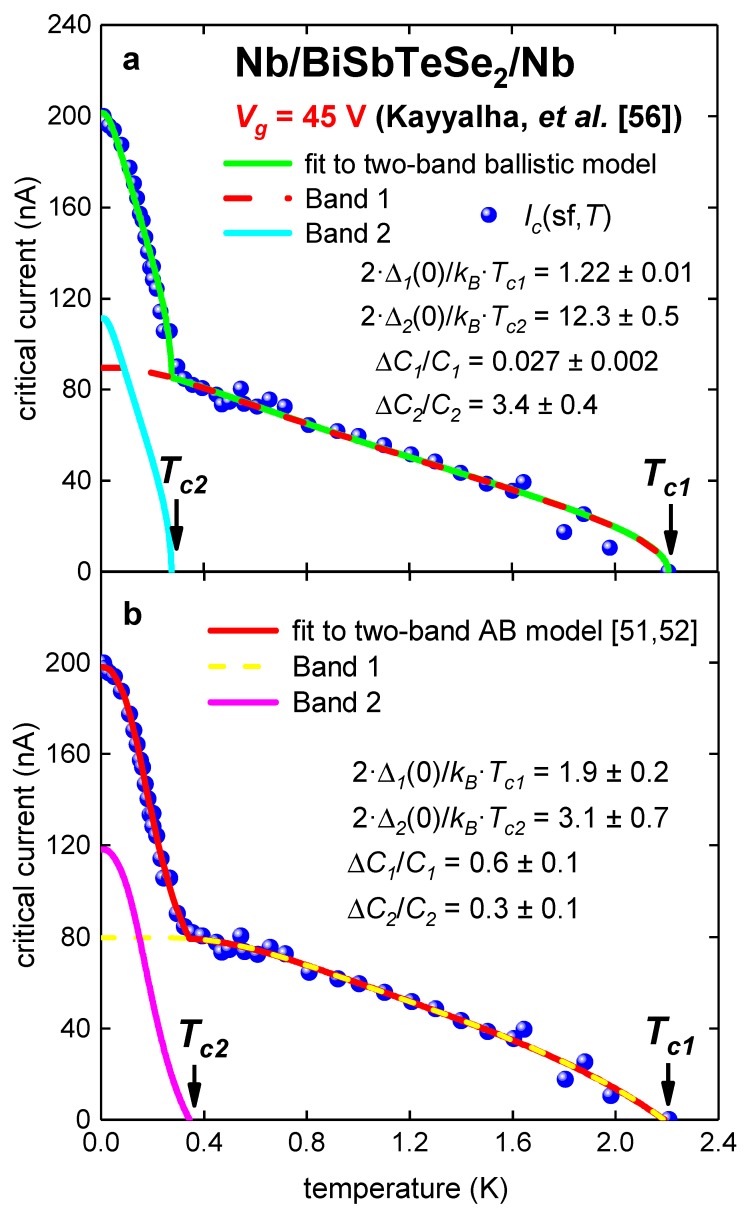
Experimental *I_c_*(sf,*T*) for Nb/BiSbTeSe_2_-nanoribbon/Nb junction (Sample 1 [56]) at gate voltage *V_g_* = 45 V. (**a**) Ballistic model, fit quality is *R* = 0.994; (**b**) modified AB model [51,52]. Derived parameters: *R_n1_* = 3.5 ± 0.3 kΩ, *R_n2_* = 630 ± 110 Ω, fit quality is *R* = 0.998.

The same conclusion can be made for Sample 1 at *V_g_* = 45 V (Figure 5 and Table 5).

### 3.3. Planar Nb/Bi_2_Se_3_/Nb Junction [56]

**Figure 6:**
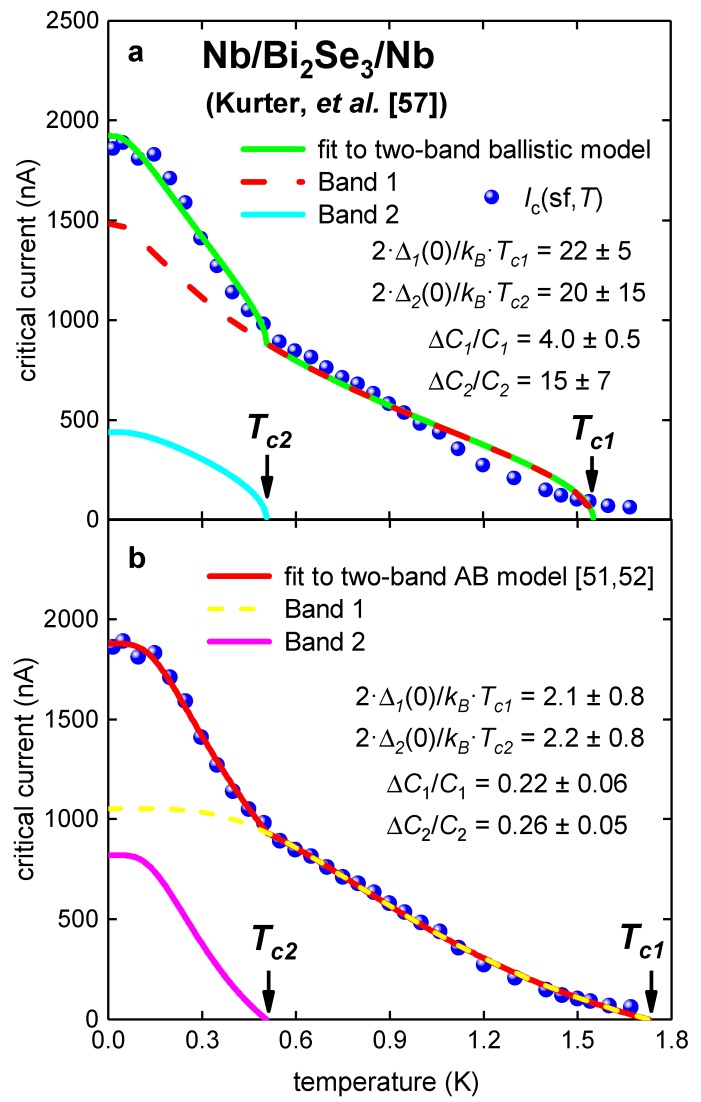
Experimental *I_c_*(sf,*T*) for Nb/Bi_2_Se_3_/Nb junction [57]. (**a**) Ballistic model, fit quality is *R* = 0.994; (**b**) modified AB model [51,52]. Derived parameters: *R_n1_* = 240 ± 100 Ω, *R_n2_* = 92 ± 33 Ω. Fit quality is *R* = 0.9991.

In Figure 6, temperature-dependent self-field critical currents, *I_c_*(sf,*T*), in Nb/Bi_2_Se_3_/Nb (*W* = 1000 nm, *L* = 100 nm) reported by Kurter et al. [57] is shown. For this junction, 300 nm < ξ*_s_* < 1,000 nm [57], and thus, the ballistic regime condition, *L* << ξ*_s_*, is well satisfied.

There is a large difference between experimental data and the fit to ballistic model (Figure 6 and Table 6). In addition, deduced parameters from the ballistic model fit have no physical interpretation. The fit to the modified AB model reveals parameters in the expected ranges (Figure 6).

There is a large difference between experimental data and the fit to ballistic model (Figure 6 and Table 6). In addition, deduced parameters from ballistic model fit have no any physical interpretation. The fit to modified AB model reveals parameters in expected ranges (Figure 6 and Table 6).

## 4. Discussion

One of the most important questions that can be discussed herein is as follows: what is the origin for such dramatic incapability of ballistic model to analyze the self-field critical currents in S/DCM/S junctions? From the author’s point of view, the origin is the primary concept of the KO theory, in that *I_c_*(sf,*T*) in the S/N/S junctions is:

(9) $$I_{c}\left(\mathrm{sf},T\right)=\underset{\varphi }{\max}\left(I\left(\varphi ,\mathrm{sf},T\right)\right)$$

where *φ* is the phase difference between two superconducting electrodes of the junction. Despite this assumption is a fundamental conceptual point of the KO theory, there are no physically background or experimental confirmations that this assumption should be a true. In fact, the analysis of experimental data by a model within this assumption (we presented herein) shows that Equation (9) is in remarkably large disagreement with experiment.

One of the simplest ways to show that Equation (9) is incorrect is to note that when the length of the junction, *L*, goes to zero, Equation (6) shows:

(10) $$I_{c}\left(\mathrm{sf},T\right)=\underset{L\to 0}{\lim}\left(1.33\cdot \frac{e\cdot \Delta \left(T\right)}{\hbar }\cdot \frac{W}{\pi \cdot L}\right)\propto \underset{L\to 0}{\lim}\left(\frac{1}{L}\right)\to \infty .$$

Herein, the simplest available function [53] that was proposed for the S/DCM/S junction in the Equation (9) was chosen as an example. However, other proposed functions for Equation (9) (for which we refer the reader to Reference [12]) have identical unresolved problem, because, as this was shown for about 100 weak-link superconductors [2,3,4,5,66], the limit should be (Equation (1)):

(11) $$I_{c}\left(\mathrm{sf},T\right)=\underset{L\to 0}{\lim}\left(1.33\cdot \frac{e\cdot \Delta \left(T\right)}{\hbar }\cdot \frac{W}{\pi \cdot L}\right)=\frac{\phi_{0}}{\pi \cdot \mu_{0}}\cdot \left[\frac{ln\left(1+\sqrt{2}\cdot \kappa_{c}\left(T\right)\right)}{\lambda_{ab}^{3}\left(T\right)}\cdot \left(\frac{\lambda_{c}\left(T\right)}{b}\cdot \tanh\left(\frac{b}{\lambda_{c}\left(T\right)}\right)\right)+\;\frac{ln\left(1+\sqrt{2}\cdot \gamma \left(T\right)\cdot \kappa_{c}\left(T\right)\right)}{\sqrt{\gamma \left(T\right)}\cdot \lambda_{ab}^{3}\left(T\right)}\left(\frac{\lambda_{ab}\left(T\right)}{a}\tanh\left(\frac{a}{\lambda_{ab}\left(T\right)}\right)\right)\right]\cdot \left(a\cdot b\right).$$

This means that the primary dissipation mechanism, which governs DC transport current limit in S/N/S, is not yet revealed. However, as we show herein, it is irrelevant to achieving values within the primary concept of KO theory, Equation (9). It should be mentioned that the Density Functional Theory (DFT) calculations [67,68] are currently unexplored powerful techniques, which can be used to reveal dissipation mechanism in S/DCM/S junctions.

## 5. Conclusions

In this paper, *I_c_*(sf,*T*) data for S/DCM/S junctions were analyzed by applying two models: the ballistic and the modified Ambegaokar-Baratoff model. It was shown that the ballistic model [10,11,12,53] cannot describe the self-field critical currents in S/DCM/S junctions. In conclusion, the ballistic model should be reexamined in terms of its applicability to describe dissipation-free self-field transport current in S/DCM/S junctions.

## Figures and Tables

**Table 1:** Deduced parameters for tantalum/graphene/tantalum (Ta/G/Ta) junction from fit to single-band Titov and Beenakker (TB) [53] and Ambegaokar and Baratoff (AB) [7,8] models.

Parameter	TB Model	AB Model
*T_c_* (K)	1.052 ± 0.002	1.06 ± 0.01
Δ*C*/*C*	17.7 ± 0.6	1.15 ± 0.07
Δ(0) (meV)	1.03 ± 0.01	0.095 ± 0.002
2·Δ(0)/*k_B_*·*T_c_*	22.7 ± 0.3	2.1 ± 0.1

**Table 2:** Deduced parameters for tantalum/graphene/tantalum (Ta/G/Ta) junction at V*_g_* = 10 V from fit to two-band Titov and Beenakker (TB) [53] and Ambegaokar and Baratoff (AB) [7,8] models.

Parameter	TB Model	AB Model
*T*_*c*1_ (K)	1.052 ± 0.001	1.053 ± 0.003
*T*_*c*2_ (K)	0.61 ± 0.02	0.63 ± 0.03
Δ*C*_1_/*C*_1_	17.1 ± 0.6	2.2 ± 0.8
Δ*C*_2_/*C*_2_	2.9 ± 3.8	1.1 ± 0.9
2·Δ_1_(0)/*k_B_*·*T*_*c*1_	21 ± 1	3.0 ± 0.9
2·Δ_2_(0)/*k_B_*·*T*_*c*2_	3 ± 1	1.9 ± 0.3

**Table 3:** Deduced parameters for Nb/BiSbTeSe_2_-nanoribbon/Nb junction (Sample 1 [56]) at V*_g_* = −20 V from fit to two-band Titov and Beenakker (TB) [53] and Ambegaokar and Baratoff (AB) [7,8] models.

Parameter	TB Model	AB Model
*T*_*c*1_ (K)	1.76 ± 0.01	1.74 ± 0.04
*T*_*c*2_ (K)	0.236 ± 0.003	0.31 ± 0.02
Δ*C*_1_/*C*_1_	0.019 ± 0.03	0.84 ± 0.18
Δ*C*_2_/*C*_2_	1.8 ± 0.3	0.19 ± 0.07
2·Δ_1_(0)/*k_B_*·*T*_*c*1_	0.83 ± 0.04	2.5 ± 0.5
2·Δ_2_(0)/*k_B_*·*T*_*c*2_	10.0 ± 0.3	2.85 ± 0.70

**Table 4:** Deduced parameters for for Nb/BiSbTeSe_2_-nanoribbon/Nb junction (Sample 1 [56]) at V*_g_* = 0 V from fit to two-band Titov and Beenakker (TB) [53] and Ambegaokar and Baratoff (AB) [7,8] models.

Parameter	TB Model	AB Model
*T*_*c*1_ (K)	2.10 ± 0.01	2.07 ± 0.03
*T*_*c*2_ (K)	0.252 ± 0.005	0.33 ± 0.02
Δ*C*_1_/*C*_1_	0.014 ± 0.001	0.6 ± 0.2
Δ*C*_2_/*C*_2_	1.5 ± 0.2	0.20 ± 0.06
2·Δ_1_(0)/*k_B_*·*T*_*c*1_	0.94 ± 0.04	1.6 ± 0.2
2·Δ_2_(0)/*k_B_*·*T*_*c*2_	9.5 ± 0.3	3.1 ± 0.7

**Table 5:** Deduced parameters for for Nb/BiSbTeSe_2_-nanoribbon/Nb junction (Sample 1 [56]) at *V_g_* = 45 V from fit to two-band Titov and Beenakker (TB) [53] and Ambegaokar and Baratoff (AB) [7,8] models.

Parameter	TB Model	AB Model
*T*_*c*1_ (K)	2.21 ± 0.01	2.19 ± 0.03
*T*_*c*2_ (K)	0.274 ± 0.006	0.34 ± 0.01
Δ*C*_1_/*C*_1_	0.027 ± 0.002	0.6 ± 0.1
Δ*C*_2_/*C*_2_	3.4 ± 0.4	0.30 ± 0.08
2·Δ_1_(0)/*k_B_*·*T*_*c*1_	1.22 ± 0.01	1.9 ± 0.2
2·Δ_2_(0)/*k*_B_·*T*_c2_	12.3 ± 0.5	3.1 ± 0.7

**Table 6:** Deduced parameters for for Nb/Bi_2_Se_3_/Nb junction [57] from fit to two-band Titov and Beenakker (TB) [53] and Ambegaokar and Baratoff (AB) [7,8] models.

**Parameter**	**TB Model**	**AB Model**
*T*_*c*1_ (K)	1.55 ± 0.02	1.73 ± 0.05
*T*_*c*2_ (K)	0.51 ± 0.03	0.51 ± 0.03
Δ*C*_1_/*C*_1_	4.0 ± 0.5	0.22 ± 0.06
Δ*C*_2_/*C*_2_	15 ± 7	0.26 ± 0.05
2·Δ_1_(0)/*k_B_*·*T*_*c*1_	22 ± 5	2.1 ± 0.8
2·Δ_2_(0)/*k_B_*·*T*_*c*2_	15 ± 7	2.2 ± 0.8
